# Embedding a Learning Management System Into an Undergraduate Medical Informatics Course in Saudi Arabia: Lessons Learned

**DOI:** 10.2196/med20.2735

**Published:** 2013-11-27

**Authors:** Nasriah Zakaria, Amr Jamal, Shekhar Bisht, Cristina Koppel

**Affiliations:** ^1^Medical Informatics and E-learning UnitMedical Education Department, College of MedicineKing Saud UniversityRiyadhSaudi Arabia; ^2^School of Computer SciencesUniversiti Sains MalaysiaPenangMalaysia; ^3^Department of MedicineImperial CollegeLondonUnited Kingdom

**Keywords:** medical education, medical informatics, learning management systems (LMS)

## Abstract

**Background:**

Public universities in Saudi Arabia today are making substantial investments in e-learning as part of their educational system, especially in the implementation of learning management systems (LMS). To our knowledge, this is the first study conducted in Saudi Arabia exploring medical students’ experience with an LMS, particularly as part of a medical informatics course.

**Objective:**

This study investigates students’ use of various features of the LMS embedded in a recently implemented medical informatics course.

**Methods:**

A mixed methodology approach was employed. Survey questionnaires were distributed to all third year medical informatics students at the end of the course. In addition, two focus group sessions were conducted with twelve students. A thematic analysis of the focus group was performed.

**Results:**

A total of 265 third year medical student surveys (167/265, 63% male and 98/265, 37% female) were completed and analyzed. Overall, 50.6% (134/265) of the students agreed that the course was well planned and up-to-date, had clearly stated objectives and clear evaluation methods, appropriate course assignment, and that the LMS offered easy navigation. Most of the students rated the course as good/fair overall. In general, females were 10.4% more likely to prefer the LMS, as revealed by higher odd ratios (odds ratio [OR] 1.104, 95% CI 0.86-1.42) compared to males. Survey results showed that students’ use of LMS tools increased after taking the course compared to before taking the course. The full model containing all items were statistically significant (χ^2^_25_=69.52, *P*<.001, n=243), indicating that the model was able to distinguish between students who had positive attitudes towards LMS and those who did not. The focus group, however, revealed that the students used social networking for general use rather than learning purposes, but they were using other Internet resources and mobile devices for learning. Male students showed a higher preference for using technology in general to enhance learning activities. Overall, medical student attitudes towards the LMS were generally positive. Students also wanted a reminder and notification tool to help them stay updated with course events. Interestingly, a subset of students had been running a parallel LMS of their own that has features worth exploring and could be integrated with an official LMS in the future.

**Conclusions:**

To our knowledge, this was the first time that an LMS was used in a medical informatics course. Students showed interest in adapting various LMS tools to enhance their learning and gained more knowledge through familiarity with the tool. Researching an official LMS also revealed the existence of a parallel student-created LMS. This could allow teacher-led and student-led platforms to be integrated in the future for an enhanced student-centered experience.

## Introduction

Around the world, medical schools are embracing e-learning technology in their curriculum. In 2011, the forum “A 2020 Vision of Faculty Development across the Medical Education Continuum” addressed how medical schools should prepare for the changing role of medical education [1], and concluded that a key focus should be the digital environment. This was based on factors such as the explosion of new information, digitization of both medical knowledge and paper-based records, students who are digital learners, and the emergence and proliferation of instructional technologies [1].

The study of e-learning focuses on the use of computer and communication technology to deliver teaching and foster learning [2]. A learning management system (LMS) is a type of software that allows educators to provide course materials and monitor, manage, and interact with students. An LMS can increase the effectiveness and efficiency of teaching in medical schools [1,3] through ease of access, better utilization of content, increased retention rate [3], cost-effectiveness [2], and learner satisfaction. In this study, it is not our intention to compare traditional and online learning approaches; rather, we want to emphasize the fact that the LMS is complementary to traditional face-to-face learning and is best used in a blended approach.

In the Kingdom of Saudi Arabia, public universities are making substantial investments in e-learning as part of their educational system. King Saud University (KSU) introduced the Deanship of e-learning and Distance Learning in 2010 [4,5]. All courses became available through the LMS via Blackboard (a commercial LMS system) in the same year. Subsequently, the College of Medicine established the medical informatics and e-learning Unit (MIELU) [6] to introduce and promote the use of e-learning among medical educators and students. Training was conducted in stages, but there was still a lack of enthusiasm among educators for full-fledged embedding of the LMS in their courses. Although most courses do now use LMS to upload lecture notes, post announcements, and deliver test grades, most do not take advantage of the other interactive tools offered by LMS, such as conferencing facilities, chat rooms, discussion boards, and evaluation tools for tests and surveys [6].

As such, our unit, MIELU, undertook an initiative to revamp the Introduction to Medical Informatics course, a third year compulsory course, in order to incorporate and exploit the full range of tools offered by the LMS. The revised course applies “blended learning”, meaning that it combines both face-to-face and online learning [2]. We injected other LMS tools such as online discussions and online quizzes to maximize the learning experience for students.

Despite the supportive environment, instructors found that integrating an e-learning approach continued to pose various challenges, despite medical students being assumed to be “digital natives”. Thus, the other important aspect of our study was to explore the extent to which Saudi students use digital technologies in their daily lives.

Prensky coined the term “digital native” to refer to people who were born into the digital era and have been exposed to computing technologies since childhood [7]. The digital native works with and around technology almost constantly; this generation may therefore be far more adaptable to e-learning technologies than the “digital immigrant,” which describes most current instructors’ generation.

A study by Jhaveri et al showed that medical students who are digital natives explore different search engines when doing coursework, use various social media to stay current with medical knowledge, and participate in blogging to promote medical discussions [3]. There is also a support from recent study among dental students that indicate they are using smartphone and tablets to learn [8]. There is an assumption that all students are of this new generation of learners and so, as they enter higher education, universities are employing online learning technologies to meet the presumed needs and expectations of these “digital natives” and enhance their learning experience [1].

Although in general there has been a shift towards e-learning within this “digital native” generation, Prensky further matured his concept to “digital wisdom” [9], described as the ability to use digital technology to complement existing abilities and decision-making. Today’s health care learners embrace online learning due to convenience and usability factors [10]. Digital wisdom de-emphasizes age and implies that it is a skill that can be learned by anyone. This is a better match to our experience in the medical informatics course, wherein students seemed to demonstrate their mastery in informatics when they have completed their final project in this course.

Implementing LMS tools in a medical informatics course is a novel study in the Gulf region because only a few medical schools here include medical informatics in their medical curriculum. The medical education curriculum environment is ever changing and evolves from year to year. Together with the e-learning tools revolution, this makes the present study highly relevant to the body of literature. The present study also clarifies areas that should be targeted in order to further promote embedding the LMS in the College of Medicine.

In this study, we investigated the use of an LMS among medical students on a medical informatics course and the issues and challenges they faced. To our knowledge, there were no previous evaluations of how well medical students are adapting to and using these tools. We also explore how our students use online tools in their daily life to better understand their translation into an educational environment.

## Methods

### Research Context: Medical Informatics Course

The introduction of medical informatics into the medical curriculum is relatively new in the Gulf region. This course was introduced as a compulsory course for medical students at KSU two years ago. Its goals are (1) to inform students about current trends in medical informatics as it applies to health care, and (2) to expand students' awareness of the ways in which information technology is used in day-to-day medical work. Two factors made medical informatics ideal to incorporate the LMS. The first is the nature of the medical informatics field itself, which involves information and communication technology; the second is that most of the instructors for this course have a strong technical background and are experienced with various e-learning tools.

**Figure 1:**
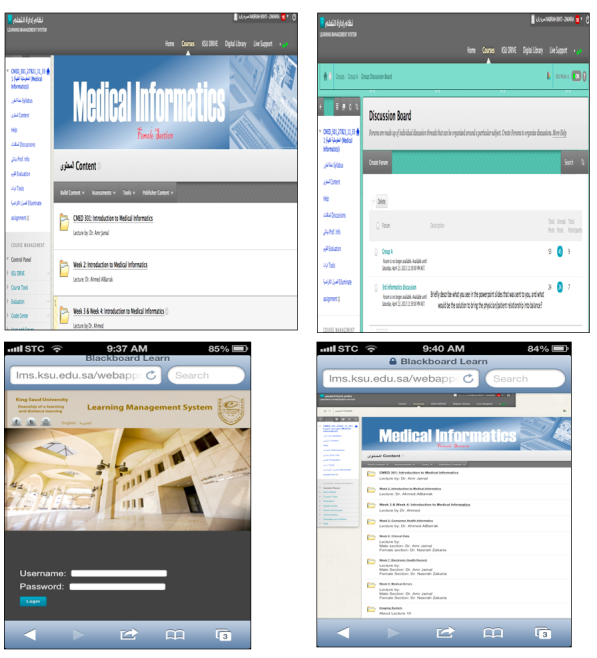
Top panels: A snapshot of LMS page for Medical Informatics course via laptop. Bottom panels: A snapshot of LMS page for Medical Informatics course via mobile device.

The course is taught through face-to-face lectures over 20 weeks. We deliver one online lecture through Flash presentation. All course materials, assignments, and quizzes are delivered via the LMS. The LMS is accessible via PC, laptop, and mobile devices (Figure 1). It is also available in both Arabic and English.

In terms of pedagogical approach, the course uses problem-based and hands-on learning. Students participate in live and online discussions, complete an article review assignment, conduct a field study, and attend workshops. We distributed 5 discussion questions (scenario- and problem-based) and students conducted small group discussions using the LMS. For one of these, the group summarizes their discussion and posts in the common forum area. This way, all groups can participate in discussions without interrupting the small group dynamics. Using the virtual class space, students were able to critically discuss pertinent topics by posting their writings, justifying their opinions, and commenting on their classmates’ ideas in a systematic manner [11]. Article review assignments were also completed on an individual basis. We posted 100 relevant medical informatics papers and each student summarized and critically assessed an article.

The LMS plagiarism checker tool was used to monitor the article review assignment. Students received instant plagiarism results, including links to similar texts found online. Online quizzes were conducted, allowing students to take the quiz anywhere within a prescribed date range and length of time. Students also had to conduct a field study project in groups. Each group visited one department or organization that used informatics and they conducted interviews to gain insight into the usage of the system and its challenges. The projects took the whole of the second semester and at the end each group presented their work formally to the instructors and invited panels from outside the university for evaluation [11]. In addition, students attended five compulsory workshops covering DxR Clinician, a Web-based simulation software for medical education, picture archiving and communication system (PACS), the hospital information system, and a mobile version of evidence based medicine.

### Survey and Statistical Analysis

We employed a mixed methodology approach, beginning with a course-wide survey of all third year students studying at the College of Medicine, KSU, Riyadh.

The survey was administered at the end of the final examination in the medical informatics course. The instrument was a structured questionnaire in English. We were not able to find similar work on LMS and medical informatics courses; however, we adapted some existing course evaluation forms. The survey included the following sections: (1) general demographic and academic information, (2) course rating, (3) perceptions about the course, (4) e-learning (LMS-Blackboard) utilization, (5) attitudes towards e-learning, and (6) proficiency in Internet/online tools. Each of the 6 sections contained 3 to 11 questions using a 5-point Likert scale (strongly disagree to strongly agree).

The survey data were collected and entered into a computer using standardized entry codes. For all tests, statistical significance was set at *P*<.05. Descriptive statistics were used to generate means, standard deviations, and percentages. In addition, *t* test (unpaired and paired) was employed to compare group variables by gender. Variables were then re-categorized into fewer groups to conduct further tests so that results could be interpreted meaningfully. We assessed the relationships of student attitudes towards e-learning using binary unconditional multiple logistic regression analysis based on gender. All the selected variables were converted into binary data (disagree/agree).

For multiple variable analyses using logistic regression, we constructed a dataset that contained only complete responses (n=243) for all relevant variables, discarding any surveys that had missing values for any of the variables involved in the regression analysis. This strategy was adopted to maintain comparability between models so that they could be developed from the same denominator. All analyses were conducted using SPSS version 21 (SPSS Inc, Chicago, IL). Logistic regression models were presented in graphical form using OpenMeta[analyst] version 4.24.13.

### Focus Group

To complement the survey, in-depth focus group sessions were conducted. Arrangements were made with student leaders to recruit 10 to 16 students in two separate sessions. The purpose of these was to investigate how students used the LMS in medical informatics; the open format allowing participants to debate the pros and cons. Focus groups also allowed us to observe the interaction among group members [12,13]. Participants were encouraged to communicate freely with each other, exchanging their experiences and commenting on each other’s stories [12,13]. Kitzinger stated that the number of focus groups can vary from 6 to 50 for a research study; however, some studies conduct only a few focus groups [12,13].

In preparation, we created a topic guide for the focus group moderator. The first author, NZ, served as moderator. The topics for discussion included issues and challenges of using the LMS, and how students overcame any struggles. We used a digital recorder to capture the focus group sessions.

The focus group session began by asking participants to fill out a form containing three questions asking them whether LMS can help achieve their educational goals, what the important skills required to use LMS are, and what challenges they faced when using the LMS. The moderator then gave a briefing on what was expected from the respondents during the focus group discussions. Next, the students discussed various interactive LMS features such as the group discussion board, online quizzes, plagiarism checker, and grade center. Students were encouraged to discuss the challenges encountered with each component and what steps they took to overcome the challenges. Thematic analysis was employed to elicit important themes that would represent the issues and challenges faced by medical students when using LMS.

## Results

### Statistical Results

**Figure 2:**
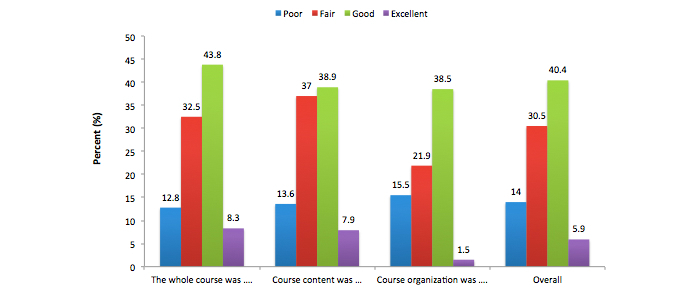
Students' perception about the course.

**Figure 3:**
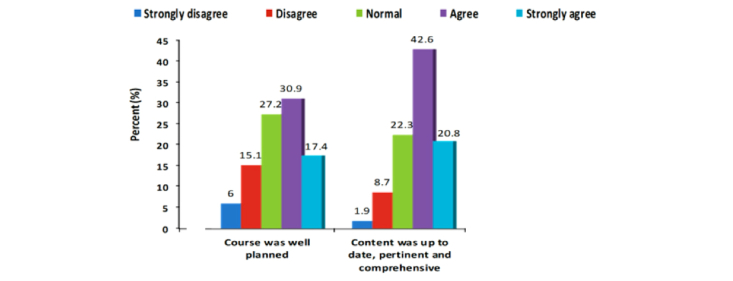
Course planning and uptodate, pertinent & comprehensiveness of content.

**Figure 4:**
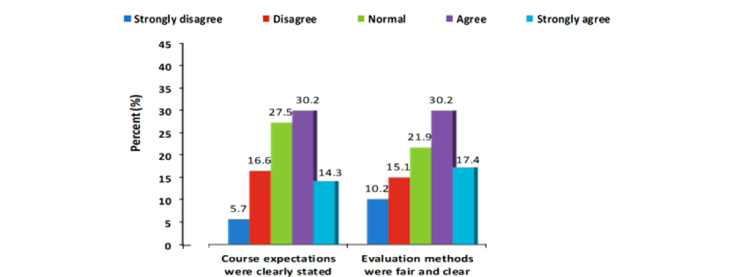
Course Expectation and evaluation methods.

**Figure 5:**
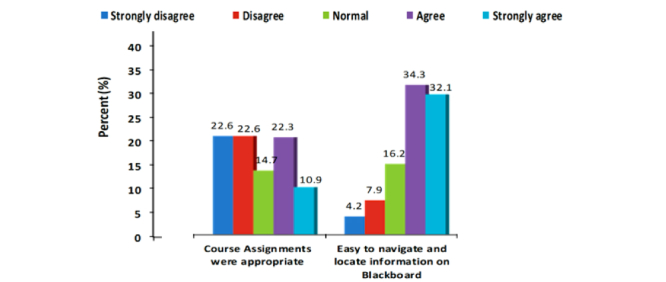
Appropriateness of course & navigation of LMS.

**Figure 6:**
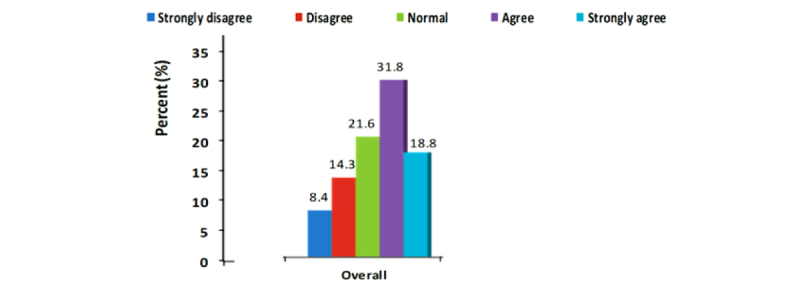
Overall perception about the course.

For this study, there were 265 third-year medical students who participated (167/265, 63.0% male and 98/265, 37.0% female). The mean age of all students was 20.3 years (SD 3.7), although mean female age (mean 21.2, SD 0.66) was significantly higher than mean male age (mean 20, SD 4.32) (Table 1). The average score achieved in the medical informatics final examination and overall cumulative GPA were 15.58 (SD 2.25) and 4.37 (SD 0.43) respectively for both genders combined (Table 1). On the other hand, female students had statistically significant higher scores in the medical informatics course (*P*<.001) as well as overall cumulative grade point average (Table 2). The student ratings indicate that about, 84.9% (225/265) found the overall course is “fair” and above. When asked about the course content, 83.8% (222/265) students agreed the content is “fair” and above (Figure 2). For the overall medical informatics course, only 14.0% (37/265) students said the course was “poor” (Figure 2). Overall, almost half of the class (50.6%, 134/265) agreed the course was well planned and up-to-date, course expectations were clearly stated, clear evaluation methods were used, course assignment were appropriate, and navigation on the LMS was easy (Figure 3-6).

Students in the present study did not show any significant difference with regard to gender or uses of the LMS except in downloading content and in sending email via the LMS. These features, however, were not frequently used (Tables 3 and 4). The online quiz was the LMS feature most often used by the students, as it was compulsory. It can clearly be observed that students’ LMS use and its incorporation into their learning significantly increased after introduction of the medical informatics course into the curriculum.

**Figure 7:**
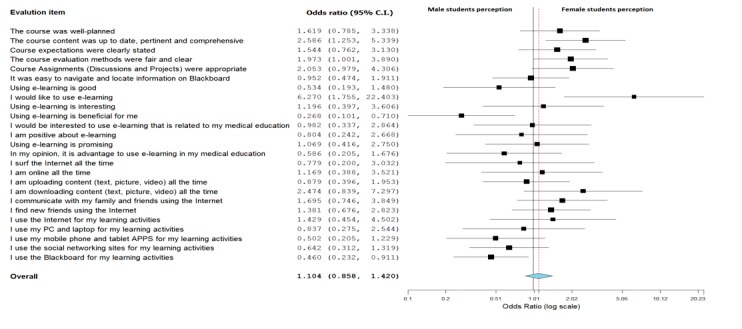
Relationships between student attitudes towards LMS and their Internet proficiency (Logistic Regression model [OR 95% CI, N=243 full case data only] modeling odds for female vs male).

In order to understand the attitudes of medical students towards LMS based on gender, we performed multiple logistic regressions. The model contained 25 independent variables (attitudes and digital natives section). The full model containing all items were statistically significant (χ^2^_25_=69.52, *P*<.001, n=243), indicating that the model was able to distinguish between students who had positive attitudes towards LMS and those who did not and correctly classified 83.7% of cases. The model is presented in Figure 7, and shows that, out of 25 items, 13 were more positively received (rated as highly agreeable) by female students as compared to male students.

The highest agreement among female students was on “using e-learning”. Analysis found that e-learning (specifically LMS-Blackboard) was 6.27 times (odds ratio [OR] 6.27, 95% CI 1.75-22.40) more likely to be used by female students as compared to male students. Similarly “course content” and “course assignments” were generally liked by female students. One interesting finding was that female students primarily used the Internet to chat with friends and family and to learn new activities other than medical education, while male students used the Internet primarily for learning activities. Male students also used Blackboard more for learning as compared to female students. Male students’ attitudes showed that they liked the e-learning (LMS) aspects of the course and reported LMS as beneficial (Figure 7).

**Table 1:** Student performance in medical informatics course.

Item	Mean (SD)	*P* value
Age	20.32 (3.73)	.012
Cumulative GPA	4.37 (0.43)	<.001
Final score in medical informatics course	15.58 (2.25)	<.001

**Table 2:** Student performance in medical informatics course by gender.

Item	Male, mean (SD)	Female, mean (SD)
Age	20.0 (4.32)	21.2 (0.66)
Cumulative GPA	4.25 (0.47)	4.5 (0.28)
Final score in medical informatics course	15.23 (5.3)	17.24 (1.63)

**Table 3:** Student use of LMS features after the medical informatics course.

Item	Mean (SD)	*P* value
Discussion board to ask questions	3.32 (1.37)	.991
Discussion board to get answers	3.16 (1.4)	.653
Safe assign to check work	3.55 (1.21)	.291
Online quizzes	4.13 (0.91)	.533
Read announcements	3.39 (1.31)	.202
Upload content (HW, Project, Papers)	3.89 (1.14)	.231
Download content (HW, Project, Papers)	3.72 (1.19)	.029
Play Flash presentation	2.35 (1.41)	.262
Send emails via Blackboard	1.77 (1.30)	.042
Receive emails via Blackboard	1.71 (1.3)	.116
Overall	3.1 (1.25)	.340

**Table 4:** Student (by gender) use of LMS features after the medical informatics course.

Item	Male, mean (SD)	Female, mean (SD)
Discussion board to ask questions	3.33 (1.45)	3.34 (1.34)
Discussion board to get answers	3.12 (1.5)	3.2 (1.34)
Safe assign to check work	3.67 (1.27)	3.5 (1.2)
Online quizzes	4.1 (0.92)	4.16 (0.92)
Read announcements	3.55 (1.3)	3.33 (1.31)
Upload content (HW, Project, Papers)	4.01 (1.16)	3.83 (1.13)
Download content (HW, Project, Papers)	3.95 (1.12)	3.61 (1.22)
Play Flash presentation	2.24 (1.4)	2.45 (1.14)
Send emails via Blackboard	1.57 (1.18)	1.91 (1.36)
Receive emails via Blackboard	1.57 (1.18)	1.91 (1.36)
Overall	3.11 (1.25)	3.12 (1.23)

### Focus Group Results

From the focus group, we found that most of the medical students reported that they used multiple resources to help with their learning. Referring to recommended textbooks was a core resource, but they also reported using search engines such as Google to familiarize themselves with the course content. When asked about the interactive tools on the LMS, the participants said it allowed them to learn new computer skills and writing skills. For computer skills, even though they reported being used to computers, they discovered new and useful tools in the LMS. They learned on their own and sought help from peers to resolve any technical issues.

In terms of writing skills, they felt that through the online discussion group they were able to write on specific topics and give comments to their peers. Students did report some technical issues when using the discussion tool, such as difficulties in creating new threads for new discussions, visibility of group members, and formatting of text in the discussion group posts. Again, they found technical solutions through their classmates. One method of getting help from their classmates was to forward screenshots of the LMS site problem to their classmates via email.

The students found the online quiz feature helped them to appreciate the LMS in their studies. They felt that the freedom to take the quiz anywhere and at any time within a particular window made it less stressful and they felt less panicked about taking quizzes. They liked the fact that there were no disturbances (eg, no proctors reminding them about the time), and reported that this helped them to complete their quizzes with ease. They also liked the fact that they were able to get their results immediately after the quiz. However, even though most of them preferred online quizzes to paper-based quizzes, they also said that when they encountered technical difficulties while taking the online quiz, they felt panicked. Another feature they liked was the PowerPoint with audio (using Flash) whereby they could view the slides multiple times and at their convenience to review the lecture content.

In general students said that after some time using the LMS, they were able to cope with this new online environment. They emphasized the importance of getting training on the system early in the semester and of the course coordinator clarifying the expectations regarding online assignments. They expressed the hope that all courses in the College will eventually use the LMS so that they can continue to adapt it into their daily academic lives. Some of them mentioned that they prefer lecturer-student communication to be done within the LMS email system so that they can better organize their learning in a single place, while others prefer using their regular email system. The one feature that they wanted was a notification service that would inform them about updates and announcements on the LMS.

Interestingly, it emerged through the focus group that some students had been running a parallel LMS of their own. The student representative would approach staff for the latest or supplementary files to upload onto an independent non-commercial server that was accessed by a subset of students to support their learning.

## Discussion

### Principal Findings

The overall attitude towards the LMS was positive among medical students in this institution. A significant odds ratio was found among the female students in regard to their preference for using the LMS. Interestingly, male students also showed high agreement in that they reported using LMS for their learning. Students were clearly using more LMS interactive tools (eg, taking online quizzes, uploading assignments, participating in discussion boards, and reading announcements) by the end of the course than before they took this course. These positive findings agree with other studies that found that an LMS was useful when introduced in medical education [8,14,15].

Significantly, medical students in Saudi Arabia were found to use mobile phones as much they were using PCs. This is consistent with previous study results, which showed that this population have access to mobile phones and find them effective for learning [15]. It also relates to the study of dental students who showed to be engaged with e-learning software using sophisticated high-end devices such as smartphones and tablets [8]. However, the present study found that students were using neither the LMS nor social networking for learning despite using the Internet, PCs, and mobile phones for this purpose. Even though these students gained digital wisdom through their use of LMS tools, we found that they take time to adapt new technologies to learning. Based on the focus group analysis, students are not as engaged in the LMS because not all courses in their medical curriculum are using the system. They would like to see all courses embedded in LMS and official communications made more readily available. Some of the students liked the idea of using email within LMS rather than their personal email. They also wanted a reminder and notification service that would update them with any news announcements, assignments and deadlines.

It is possible that the positive attitude of medical students in embracing LMS was due to the fact that the course content itself included various technologies that will help them in their medical careers. For example, electronic health records, clinical decision support systems, and computerized physician order entry, all of which are technologies that have been shown to increase efficiencies in health care. In addition, during the learning process, the instructors in this course (authors AJ and NZ) applied various technologies such as Flash video and e-voting to capture students’ attention to the subject matter.

The discovery of a parallel student-run LMS is not surprising, given the abilities of our digitally wiser students. Described as “Edupunk”, using free technology to address specific needs has previously been incorporated into large university environments [16]. In addition, prior studies also indicate that students prefer to have online repositories for efficient access to learning resources [10]. This is an exciting opportunity for further research work to explore by us. Exploring which features students are duplicated as well as identifying additional LMS features will give more insight into their utility and student preferences. The teacher-led and student-led LMSs could also be integrated in the future in order to better serve all students. This would also enable the more digitally wise students to contribute to the delivery of the course and expand their skills, thus providing a more student-centered approach and maximizing learning across students of varying ability.

Graz University in Austria propose a combined Student Centered e-Learning (SceL) approach where students explore e-learning tools by themselves in a supportive enviroment [17]. Through this, students have been shown to gain “personal values” such as flexibility, self-confidence, and social skills. They present a case study of a computer science course to highlight how both students and teachers gain from SceL. The researchers emphasize “personalization” and “creativity” as the important ingredients for the LMS. Personalization focuses on user needs while creativity allows educators to explore new pedagogical approaches [16].

### Limitations

First, the study was conducted in only one medical college, though it is a well-reputed medical college in the middle-eastern region and includes students from all of Saudi Arabia as well as regional students. This could be considered a limitation. Second, due to the lack of related studies and standardized surveys on this topic, we had to design our own questionnaire. Since this is the first time the questionnaire has been used, that could also be considered a limitation. The questionnaire could be expanded to yield a more precise evaluation of student attitudes, perception, and feedback regarding e-learning and LMS .

Another aspect that we did not explore in this research is the extent to which students have been previously exposed to technology [18]. We did not measure what other devices they may be familiar with and how this impacts LMS usage. Holzinger et al [18] describe how elderly users are able to accept new technology when they can relate to it through metaphors and to technology they have previously been exposed to.

### Conclusions

The present study's findings indicate that most of the students found the medical informatics course to be organized and has good content. Female students preferred this course more strongly than male students. Overall, we found students were successful in adapting various learning technologies and continuously experimenting to make better utilization of the LMS for their learning. This includes some students using their own online tools to maintain a parallel LMS. In future, we expect students will expand their use of the LMS when all medical courses are fully integrated in LMS. We intend to further investigate the student-led LMS in order to optimize the tools we offer our students and involve them in their delivery for an enhanced student-centered experience.

## Abbreviations

DxR: Diagnostic Reasoning
KSU: King Saud University
LMS: Learning Management System
MIELU: medical informatics and e-learning Unit
OR: odds ratio
PACS: picture archiving and communication system
SceL: student centered e-learning
